# A Protocol for the Management of the Inpatient Fracture Neck of Femur is Required

**DOI:** 10.2174/1874325001812010358

**Published:** 2018-08-31

**Authors:** Carl Malcolm Green, Nikhil Shah

**Affiliations:** 1 Cavendish Hip Fellow, Sheffield Teaching Hospitals NHS Trust, Northern General Hospital, Herries Road, Sheffield, S5 7AU, England; 2 Consultant Orthopaedic Surgeon, Wrightington Hospital, Hall Lane, Wigan, WN6 9EP, England

**Keywords:** Inpatient, Fracture, Neck of femur, Protocol falls, Surgery, NHFD

## Abstract

Falls within a hospital environment are a major cause of morbidity and may even lead to mortality. Pathways for patients suffering a Fractured Neck of Femur (FNOF) in the community are well established following the development of the Blue Book, BOAST guidelines and National Hip Fracture Database (NHFD). However, there is no such agreed pathway for patients suffering FNOF within a hospital environment. Such patients have been demonstrated to have a higher risk of delays in medical optimisation, delays in operative management, and mortality. There is, therefore, a need to create a nationally agreed guideline for the care of the “inpatient FNOF” as this is an important subgroup of patients. This article highlights this issue as well as advising medical staff on how to identify a potential FNOF within a hospital environment in order to ensure prompt management of a vulnerable group of patients.

## 1 INTRODUCTION

Falls within hospital are a major cause of morbidity and may even lead to mortality. Approximately 240,000 falls are recorded within the National Health Service (NHS) every year in England and Wales [1] and this costs the NHS approximately £2.3 billion per year [2]. As 152,000 falls were registered in 2007, this is an increasing problem within an ageing population [3]. This is being addressed by the use of the National Falls Audit, updated guidelines from the National Institute for Health and Care Excellence (NICE) in 2013, and increasing education and awareness of falls prevention.

Falls can also lead to an “inpatient” Fracture Neck of Femur (FNOF). According to the National Hip Fracture Database (NHFD), 2,511 (3.9% of all hip fractures) patients in 2015 and 2,659 patients (4.1%) in 2016 were treated for an inpatient fracture, meaning that this is an increasing problem [4, 5]. Other research performed in individual hospital trusts report that approximately 5.5 - 6.1% of hip fractures occur following a fall within a hospital environment [6-8]. Although the higher incidence of fracture in these studies can be explained by characteristics of the trusts (as two units included injuries from rehabilitation centres within their trusts), another reason may be the reliability of NHFD data. In one study, three of 40 patients who suffered a fracture within a hospital environment had been listed as suffering the injury within their “own home” according to the data submitted to the NHFD [6].

Protocols for the management of patients admitted from the community with an acute FNOF are well established following the publication of the Blue Book and BOAST guidelines, the use of the Best Practice Tariff (BPT) and the development of the National Hip Fracture Database (NHFD) [4, 5, 9, 10]. NICE guidelines, last updated in 2017, also provide recommendations for multidisciplinary management of patients suffering this injury [11].

Standard care for a patient with a suspected FNOF now includes multidisciplinary team care, adequate analgesia and hydration, urgent Xrays and transfer to the orthopaedic unit, surgery within 36 hours, and orthogeriatrician review within 72 hours. MRI or CT scans are required in symptomatic patients within 24 hours of injury if Xrays fail to diagnose this injury. Mental state examinations, falls assessments and bone health assessments also form part of this pathway [11].

However, although these parameters are applied when treating the “inpatient” FNOF, there is no set protocol for this group of patients. There is no guidance as to whether the time of fall or the time at which the injury is diagnosed should be when the clock is started in order to ensure prompt treatment, nor are there agreed protocols for the immediate post-falls care of these patients or incident reporting [12]. This is important as such patients have been shown to have a higher level of impaired mental status, co-morbidity (including respiratory, renal, endocrine and malignant pathologies) [7, 13], and risk of postoperative mortality of 35.7 - 50% within 1 year compared to 21.5 – 31.2% those falling in the community [6-8]. Compensation has also been paid to patients following an inpatient fall resulting in FNOF [14, 15] hence this is becoming increasingly important from a medicolegal perspective.

Falls prevention and optimising medical care of vulnerable patients is therefore a key step in this process. Patients who fracture their neck of the femur as an inpatient have commonly fallen at least once prior to their injury [12], hence active fall prevention strategies are required as these have been shown to reduce the incidence of falls [16]. Recommendations following the National Falls Audit in 2015 state that all patients over 65 years of age (or over 50 with a condition affecting their mobility) should be considered as a falls risk as opposed to conducting falls scores [1].

Post-falls care is also vitally important. Pathways for patients following a fall may vary between hospital trusts but all should include an assessment for head injury or fracture, safe manual handling of patients with a suspected injury, and easy access to post-fall protocols, specialist equipment, and relevant investigations and treatment. Should a patient suffer a fall, an immediate assessment by ward staff is required in order to identify any injuries to the patient’s head, spine or extremities. If there is any suspicion of a significant lower limb fracture, patients should be transferred to bed using a spinal board, “scoop” or similar device [12] and be promptly examined by a medical practitioner.

**Fig. (1):**
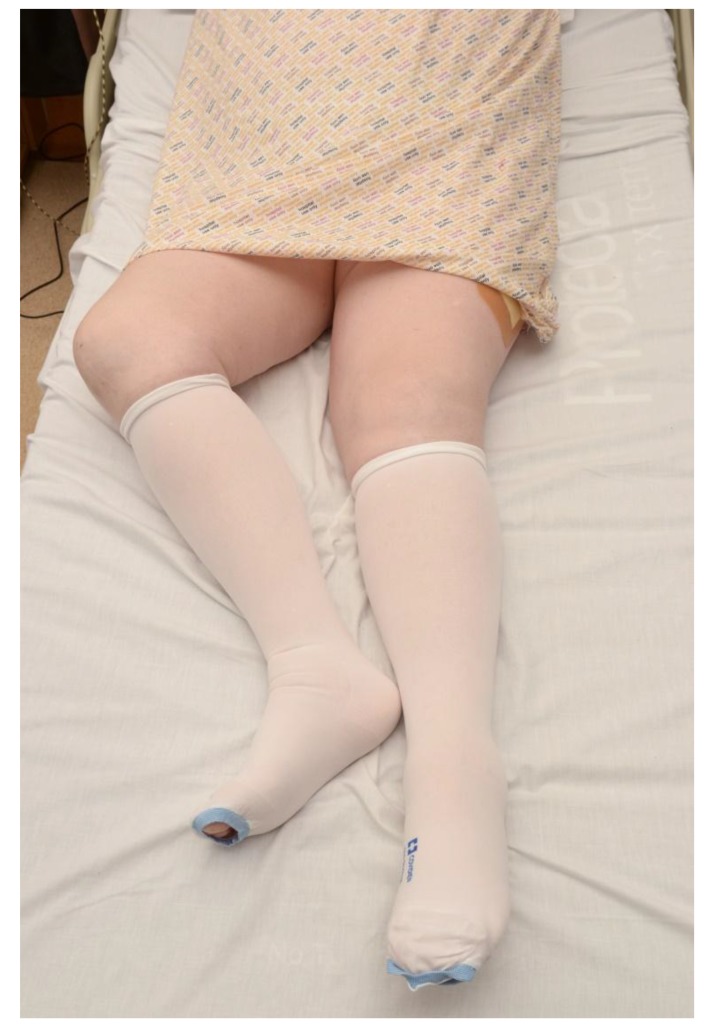
A displaced FNOF shows a shortened, externally rotated lower limb.

Examination of a patient with a suspected FNOF should be performed in the same manner within a ward setting as it would be in an emergency department. Examination of the hip should be done in a step-wise manner and starts with assessing patient leg lengths; a displaced FNOF shows a shortened, externally rotated lower limb (Fig. **1**).

**Fig. (2):**
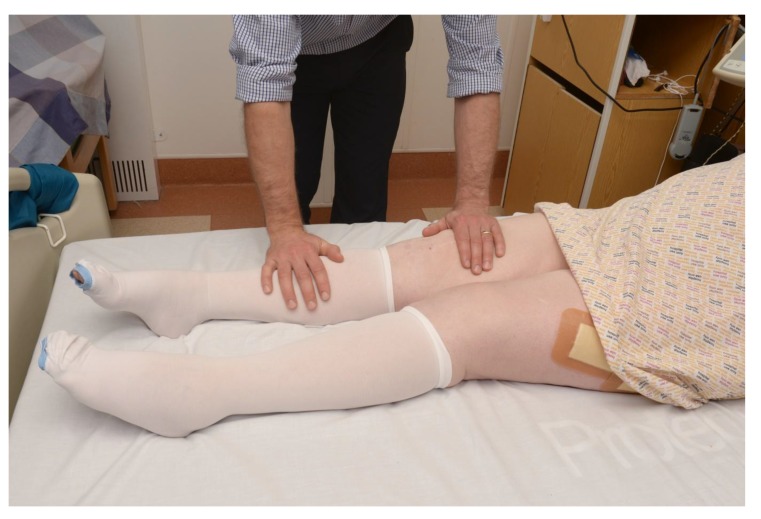
“Pin-rolling” of the affected leg to assess for pain.

If the leg adopts a normal position when supine, perform a gentle “pin-rolling” of the affected leg to assess for pain (Fig. **2**).

**Fig. (3):**
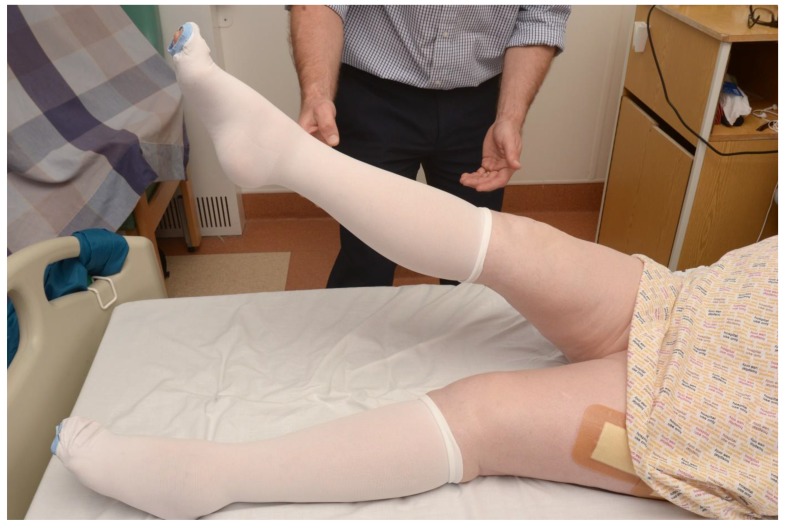
Assess patient's ability to perform a straight leg raise test.

If the patient does not complain of pain, ask the patient to lift their leg off the bed as failure to do so may indicate a hip fracture (Fig. **3**).

**Fig. (4):**
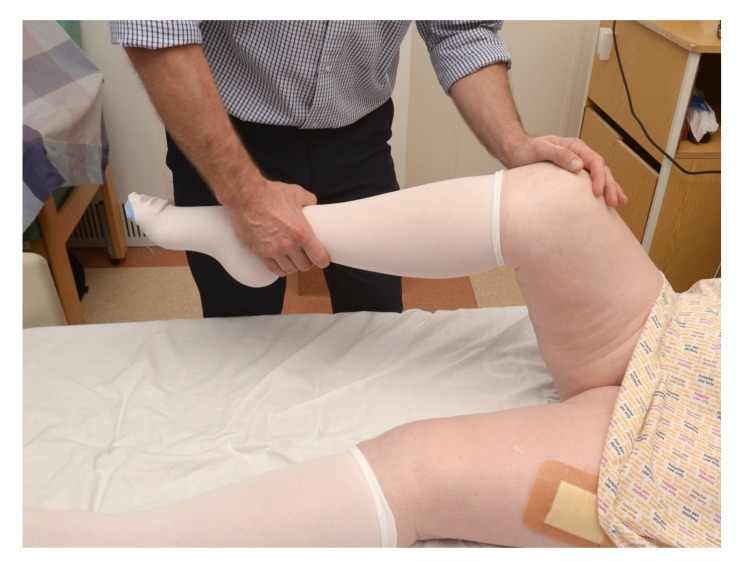
Internal and external rotation of the hip.

Finally, if this can be done, assess their range of motion in flexion, abduction, and internal and external rotation of the hip (Fig. **4**).

If at any stage the patient complains of pain within the hip and groin during these steps, stop the examination and obtain urgent Xrays (AP Pelvis and Lateral Hip) to assess for fracture. If no fracture can be identified but the patient remains symptomatic, further imaging will be required by MRI scan within 24 hours of injury, or CT scan if MRI is contraindicated or unavailable [10].

If a fracture is confirmed, early operative intervention is required as it is well established that mortality among all groups of patients suffering an FNOF improves with early intervention [17-19]. Urgent pre-operative transfer to an orthopaedic ward is therefore advised unless this would be detrimental to the patient from a medical perspective (*e.g*. they are on a specialist ward for their presenting complaint). Delays in operative management have also been shown to lead to an increased risk of deep vein thrombosis [20]. An early assessment by a specialist Orthogeriatrician, focussing on optimising medical issues which would otherwise delay operative management, is therefore essential as this has been demonstrated to improve patient outcome [21, 22]. As a result of their co-morbidities, inpatients suffering hip fractures are more likely to have their procedures postponed due to medical issues, or have a significant delay in their diagnosis either due to imaging not being performed in a timely fashion or because further imaging is required [6, 13].

In order to gain a full picture as to how this injury impacts on inpatients, further measures should be taken. The time of injury (*i.e*. the exact time of falling) should be used as the start time for the 36-hour time limit for operative intervention and all examinations and investigations for these patients in order to prepare them for operative intervention must be treated as a priority. An incident report should be completed and all confirmed FNOF injuries are to be rated as “severe” according to guidance from the National Patient Safety Agency as this is a life-changing or even a life-threatening injury [3]. A Root Cause Analysis (RCA) should be performed and disseminated within the involved ward and department in order to learn lessons from the incident in an effort to reduce the likelihood of other patients suffering a similar injury [12].

Although NICE guidance did not specifically include the inpatient fracture as a separate issue when updated in 2017, the latest update in the NHFD did include a recommendation that hospital managers and clinicians examine how ward environments and staffing contribute towards inpatient falls and take steps to minimise risk [5, 11]. The authors of this article therefore request that nationally agreed guidance is created for patients suffering an “inpatient” fracture neck of the femur with a view to be included in the next updated version of NICE guidance.

## CONCLUSION

Exact protocols for falls prevention and post-fall care may vary between hospital trusts in the NHS due to the variability of regional parameters, resources, and patient demographics. However, key themes should be agreed. In summary, the authors would suggest using the time of injury as the starting point for all patients with a suspected inpatient FNOF, that patients are treated as a priority and transferred to an orthopaedic trauma ward if medically fit to do so. Management of their injury should then follow NICE guidelines and the BPT, with the aim to intervene surgically within 36 hours of the injury. All confirmed FNOF injuries should be treated as a “severe” injury on incident reporting and a full root cause analysis should be performed in order to minimise the risk of future inpatient injury, especially due to medicolegal issues surrounding this type of injury highlighted within this report. Finally, outcome measures of inpatient FNOF injuries should be assessed separately to community FNOF injuries due to the knowledge of an inferior outcome within this group of patients.

## References

[1] (2015). Falls and Fragility Fracture Audit Programme (FFFAP). National Audit of Inpatient Falls 2015..

[2] National Institute of Clinical Excellence (2013). Falls in older people: assessing risk and prevention. Clinical guideline [CG161].

[3] National Patient Safety Agency (2007). Slips, trips and falls in hospitals..

[4] (2016). National Hip Fracture Database Report.

[5] (2017). National Hip Fracture Database Report.

[6] Green C.M., Zeiton M., Foulkes K., Barrie J. (2014). The inpatient fracture neck of femur: An important subgroup of patients.. Injury.

[7] Johal K.S., Boulton C., Moran C.G. (2009). Hip fractures after falls in hospital: A retrospective observational cohort study.. Injury.

[8] Mohamed M., Patel D., Zhao S., Ballal M.S., Scott S. (2015). Increased mortality amongst inpatients sustaining neck of femur fractures as in-patients in a Trauma centre.. Open Orthop. J..

[9] British Orthopaedic Association and British Geriatrics Society (2007). The care of patients with fragility fractures (“Blue Book”).

[10] British Orthopaedic Association (2012). Standards for Trauma: Patients sustaining a fragility hip fracture (BOAST 1, version 2).

[11] (2011). NICE Guidelines for the Care of Fracture Neck of Femur (CG124). National Institute of Clinical Excellence, 2011 (Updated April 2017).

[12] Green C.M., Zeiton M., Foulkes K., Barrie J. (2015). The inpatient fracture neck of femur: Severe injuries which need to be taken seriously.. J Patient Saf..

[13] Foss N.B., Palm H., Kehlet H. (2005). In-hospital hip fractures: Prevalence, risk factors and outcome.. Age Ageing.

[14] Simpson millar solicitors £12,000 compensation awarded in hospital fall.

[15] Hudgell Solicitors £17000 compensation secured for hospital patient after broken hip was missed by doctors.

[16] Stenvall M., Olofsson B., Lundström M., Englund U., Borssén B., Svensson O., Nyberg L., Gustafson Y. (2007). A multidisciplinary, multifactorial intervention program reduces postoperative falls and injuries after femoral neck fracture.. Osteoporos. Int..

[17] Bottle A., Aylin P. (2006). Mortality associated with delay in operation after hip fracture: Observational study.. BMJ.

[18] Bretherton C.P., Parker M.J. (2015). Early surgery for patients with a fracture of the hip decreases 30-day mortality.. Bone Joint J..

[19] Nyholm A.M., Gromov K., Palm H., Brix M., Kallemose T., Troelsen A. (2015). Time to surgery is associated with thirty-day and ninety-day mortality after proximal femoral fracture: A retrospective observational study on prospectively collected data from the danish Fracture database collaborators.. J. Bone Joint Surg. Am..

[20] Smith E.B., Parvizi J., Purtill J.J. (2011). Delayed surgery for patients with femur and hip fractures-risk of deep venous thrombosis.. J. Trauma.

[21] Gosch M., Hoffmann-Weltin Y., Roth T., Blauth M., Nicholas J.A., Kammerlander C. (2016). Orthogeriatric co-management improves the outcome of long-term care residents with fragility fractures.. Arch. Orthop. Trauma Surg..

[22] Kristensen P.K., Thillemann T.M., Søballe K., Johnsen S.P. (2016). Can improved quality of care explain the success of orthogeriatric units? A population-based cohort study.. Age Ageing.

